# No increase of serum neurofilament light in relapsing-remitting multiple sclerosis patients switching from standard to extended-interval dosing of natalizumab

**DOI:** 10.1177/13524585221108080

**Published:** 2022-07-20

**Authors:** Magnus Johnsson, Helen H Farman, Kaj Blennow, Henrik Zetterberg, Clas Malmeström, Markus Axelsson, Jan Lycke

**Affiliations:** Department of Clinical Neuroscience, Institute of Neuroscience and Physiology, Sahlgrenska Academy, University of Gothenburg, Gothenburg, Sweden/Sahlgrenska University Hospital, Gothenburg, Sweden; Department of Clinical Neuroscience, Institute of Neuroscience and Physiology, Sahlgrenska Academy, University of Gothenburg, Gothenburg, Sweden; Clinical Neurochemistry Laboratory, Sahlgrenska University Hospital, Mölndal, Sweden/Department of Psychiatry and Neurochemistry, Institute of Neuroscience and Physiology, Sahlgrenska Academy, University of Gothenburg, Mölndal, Sweden; Clinical Neurochemistry Laboratory, Sahlgrenska University Hospital, Mölndal, Sweden/Department of Psychiatry and Neurochemistry, Institute of Neuroscience and Physiology, Sahlgrenska Academy, University of Gothenburg, Mölndal, Sweden/Department of Molecular Neuroscience, Institute of Neurology, University College London, London, UK/UK Dementia Research Institute, University College London, London, UK; Department of Clinical Neuroscience, Institute of Neuroscience and Physiology, Sahlgrenska Academy, University of Gothenburg, Gothenburg, Sweden; Department of Clinical Neuroscience, Institute of Neuroscience and Physiology, Sahlgrenska Academy, University of Gothenburg, Gothenburg, Sweden; Department of Clinical Neuroscience, Institute of Neuroscience and Physiology, Sahlgrenska Academy, University of Gothenburg, Gothenburg, Sweden

**Keywords:** Multiple sclerosis, biomarkers, neurofilament light, natalizumab, extended dosing interval, MRI, EDSS

## Abstract

**Background:**

Accumulating evidence supports the efficacy of administering natalizumab (NZ) with extended-interval dosing (EID) in patients with relapsing-remitting multiple sclerosis (RRMS).

**Objectives:**

We switched NZ dosing from 4-week to 6-week intervals in patients with RRMS, and investigated the effect on serum neurofilament light chain (sNfL) concentrations.

**Methods:**

We included two cohorts of patients with RRMS treated with NZ: one received the standard-interval dosing (4 weeks) at baseline, and were switched to 6-week intervals (EID4–6, *N* = 45). The other cohort received EID (5- or 6-week intervals) both at baseline and during follow-up (EID5/6, *N* = 25). Serum samples were collected in the EID4–6 cohort at every NZ infusion, for 12 months. The primary outcome was the change in sNfL concentrations after switching to EID.

**Results:**

The baseline mean sNfL concentration in the EID4–6 cohort was 10.5 ng/L (standard deviation (SD) = 6.1), and it remained unchanged at 12 months. Moreover, individual sNfL concentrations did not change significantly after extending the NZ dosing intervals. In addition, the EID4–6 and EID5/6 cohorts had similar baseline sNfL concentrations.

**Conclusion:**

We concluded that extending the NZ dosing interval did not increase axonal damage, as determined with sNfL, in patients with RRMS.

## Introduction

Natalizumab (NZ) is a monoclonal antibody used for treating patients with relapsing-remitting multiple sclerosis (RRMS).^1^ NZ is administered according to a standard dosing schedule of 300 mg every 4 weeks (standard-interval dosing; SID). When NZ binds to α4 integrin on the surface of leukocytes, it prevents leukocyte migration from the blood into the central nervous system (CNS). NZ effectively reduces disease activity in RRMS, and it is well tolerated with a few adverse effects.^2^ The main drawback of NZ is that it increases the risk of progressive multifocal leukoencephalopathy (PML),^3^ a John Cunningham (JC virus) virus infection that often gives rise to severe impairment and is lethal in 24% of patients treated with NZ.^4^ In non-randomized observational studies, extended-interval dosing (EID) was associated with a significantly lower risk of developing PML, compared to SID,^5^ but it had similar therapeutic efficacy.^6–9^ Very recently, therapeutic efficacy has also been demonstrated to be maintained in EID with NZ in a randomized controlled study.^10^

Previously, when switching NZ dosing from SID to EID, disease activity and progression was generally monitored with conventional cerebral magnetic resonance imaging (MRI) and clinical evaluations.^6,7,9,11^ However, current evidence has suggested that signs of inflammatory activity and neurodegeneration may escape detection with conventional monitoring.^12–14^

The most promising soluble biomarker in MS is neurofilament light (NfL),^15^ a marker of axonal damage that can be determined in cerebrospinal fluid (CSF)^16^ as well as in blood.^17,18^ There is accumulating evidence that NfL is a reliable biomarker of disease activity in RRMS,^19,20^ that may also reflect therapeutic efficacy.^21^ Consequently, NfL has served as an additional outcome measure in clinical trials.^22^

This study aimed to determine whether switching NZ treatment intervals from SID to EID might affect serum neurofilament light chain (sNfL) concentrations in patients with RRMS. To reduce potential effects of other factors on sNfL levels, we selected patients who lacked signs of disease activity in clinical and MRI examinations.

## Material and methods

### Study design and patients

**Figure 1.**
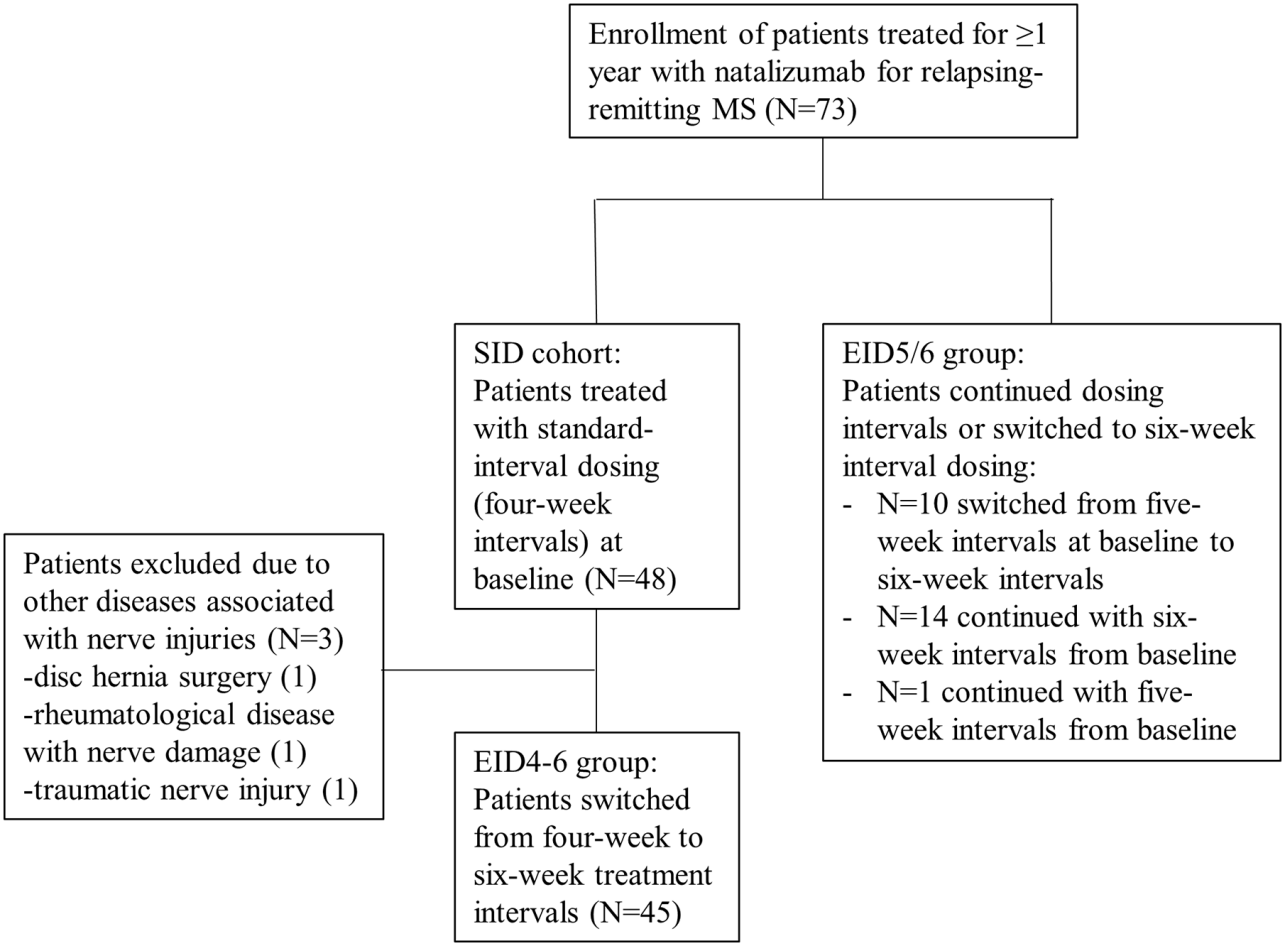
Flow chart of patient selection and treatment allocation.

This prospective observational single-center study was conducted for 12 months at the MS center, Sahlgrenska University Hospital in Gothenburg, Sweden. Eligible patients had RRMS, fulfilled the 2017 McDonald criteria,^23^ and had been receiving 300 mg NZ (Tysabri^®^, Biogen, Cambridge, MA, USA) intravenously, every 4, 5, or 6 weeks, for at least 1 year. They should not have any relapse or new or enlarging lesions on MRI within 6 months prior to baseline. After signing informed consent forms, patients were consecutively enrolled in the study. The first patients were included on the 1st of Oct 2019 and the last follow-up visit was on the 1st of June 2021. The inclusion process is illustrated in Figure 1.

### Procedures

The study participants were divided into two cohorts, based on the NZ infusion interval; one cohort had received NZ at 4-week intervals prior to baseline, and they were switched to receive EID at 6-week intervals (EID4–6). The other group had received EID at 5- or 6-week intervals at baseline, and continued extended dosing (EID5/6). Except for one patient, the patients in EID5/6 who received EID at 5-week intervals at baseline switched to 6-week intervals.

**Figure 2.**
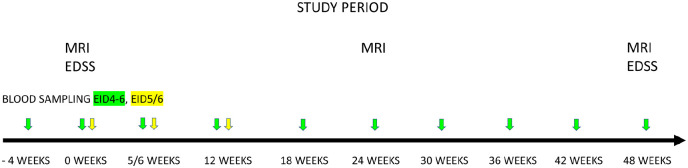
Study design for testing the effects of extended natalizumab dosing in patients with relapsing-remitting MS. Patients in the EID4–6 treatment group (green) switched from standard (4-week) to extended (6-week) dosing intervals; patients in the EID5/6 group (yellow) remained on extended dosing intervals (i.e. 5- or 6-week intervals). Blood samples were drawn (color-coded arrows) to analyze serum neurofilament light chain concentrations. Patients underwent conventional monitoring with MRI and EDSS at the indicated times. EDSS: Expanded Disability Status Scale.

In the EID4–6 cohort, peripheral blood was drawn at 4 weeks prior to baseline, at baseline (week zero), and then every 6 weeks, up to 48 weeks. In the EID5/6 cohort, blood was drawn at baseline (week zero), at 5/6 weeks, and at 12 weeks. In both study cohorts, MRI scan was performed at baseline, at 24 weeks, and at 48 weeks, and clinical examination with Expanded Disability Status Scale (EDSS)^24^ scoring were performed at baseline and at 48 weeks (Figure 2).

Baseline demographic and clinical characteristics were retrieved from the Swedish MS Registry (SMSreg, http://www.msreg.net) and from electronic patient records. A relapse was defined as the appearance of new or worsening neurological symptoms compatible with MS that lasted more than 24 hours in the absence of any factor that could cause worsening of symptoms (i.e. a pseudo relapse). Disability was assessed at clinical visits or via telephone due to the COVID-19 pandemic, and scored with EDSS or telephone-EDSS.^25^ Significant disability progression was defined as an increase of 1.5 points from a baseline EDSS score of 0, an increase of 1 point from a baseline EDSS score of 1–5.5, and an increase of 0.5 point from a baseline EDSS score >5.5. The MRI protocol included the brain and cervical spinal cord with T1-weighted images, T1-weighted images with gadolinium contrast, T2-weighted images, fluid-attenuated inversion recovery (FLAIR) images, and diffusion-weighted imaging (DWI), performed according to the Swedish guidelines.^26^ No evidence of disease activity (NEDA-3) was defined as a lack of relapse, no new or enlarging lesions detected on MRIs, and no significant progression during the study period.^27^

Intravenous peripheral blood samples were obtained prior to NZ infusion. Samples were collected in three pairs of 5-mL serum-gel and plasma containers. Serum samples were maintained at room temperature for 30 minutes to allow complete clotting. The samples were spun at 2000*g* for 10 minutes, then aliquoted in 1-mL portions and frozen directly at −80°C.

All NfL analyses were performed by board-certified laboratory technicians who were blinded to clinical data. To minimize variation, baseline and follow-up samples were analyzed side-by-side on each assay plate using one batch of reagents. In addition, samples from healthy controls were randomly analyzed in each assay plate. All analyses were performed at room temperature. Serum NfL concentration was measured using the Simoa^®^ NF-light™ Advantage Kit on an HD-X Analyzer (Quanterix, Billerica, MA, USA). Briefly, the samples, including internal quality control samples, and calibrator stock were removed from storage and allowed to thaw at room temperature. The RGP reagent was shaken for 30  minutes at 800  r/min and heated to 30°C. The calibrators, samples, and QCs were vortexed for 30  seconds at 2000  r/min. The internal calibrators, samples, and QCs were additionally centrifuged for 10  minutes at 4000*g*. Calibrators, samples, and QCs were added to the plate and covered with sealing tape. Reagents, samples, and calibrators were run in the HD-1 Analyzer using a 4× dilution. The intra-assay and inter-assay coefficients of variation were 10%.

### Standard protocol approvals, registrations, and patient consents

This study was conducted in accordance with the Declaration of Helsinki and the International Good Clinical Practice guideline. The study was approved by the Regional Committee for Medical Research Ethics, Gothenburg (EPN-460-13) and the Swedish Ethical Review Agency (DNR 2020-04900). Written informed consent was obtained from all participating patients.

### Statistical analyses

Statistical analyses were performed with SAS 9.4 (SAS Institute Inc., Cary, NC, USA), SPSS version 23 (IBMCorp., Armonk, NY, USA), and GraphPad Prism 9.3 (GraphPad Software, San Diego, CA, USA). For continuous variables, comparisons between groups were performed with Fisher’s Non-Parametric Permutation Test.^28^ For matched pairs, comparisons within groups were performed with Fischer’s Non-Parametric Permutation test. Analysis of covariance (ANCOVA) was performed to adjust for age. The Wilcoxon signed-rank test was performed to evaluate differences between sNfL and plasma neurofilament light chain (pNfL) and changes in EDSS from baseline to 12 months. Pearson’s correlation coefficients were used to evaluate correlations between sNfL and age or body mass index (BMI). Pearson’s correlation analysis and the Shrout-Fleiss reliability random test^29^ were performed to compare sNfL and pNfL. The Mersenne Twister was used for random number generation.

## Results

The study included 73 patients with RRMS; 48 had been treated with SID and 25 had been treated with EID. Three patients in the EID4–6 cohort were excluded due to concomitant conditions with neurological injuries (Figure 1). The demographic and clinical characteristics of the study cohorts are presented in Table 1.

**Table 1.** Baseline demographic and clinical characteristics of patients with RRMS.

Characteristics	EID4–6	EID5/6
Patients, *N*	45	25
Sex, female/male; *N* (%)	40/5 (89%/11%)	17/7 (68%/22%)
Age, years	43 (25–73)	45 (23–61)
BMI, kg/m^2^	24.6 (16–48.3)	27 (20.6–56.2)
Median EDSS score	2 (2.0; 0–4.5)	2 (2.1; 0–6.5)
Disease duration, years	13.4 (3–42)	11 (2–27)
NZ treatment, years	5 (1–11)	5.8 (1–12)
Interval from previous MS relapse to baseline, years	7.4 (1–17)	6.4 (2–16)
DMTs before NZ	1.8 (0–4)	1.5 (0–2)
Patients treated with 4-week SID, *N*	45	0
Patients treated with 5-week EID, *N*	0	11
Patients treated with 6-week EID, *N*	0	14
JC virus antibody positivity, *N*	0	18

RRMS: relapsing-remitting multiple sclerosis; EID: extended-interval dosing; EID4–6: patients switched from treatment at 4-week intervals to treatment at 6-week intervals; EID5/6: patients treated at 5- or 6-week intervals; BMI: body mass index; EDSS: Expanded Disability Status Scale; NZ: natalizumab; MS: multiple sclerosis; DMT: disease-modifying treatment; SID: standard-interval dosing; JC virus: John Cunningham virus.

Values are the mean (range), unless indicated otherwise.

One patient in the EID5/6 cohort experienced a relapse and a new non-enhancing lesion was detected on MRI. However, none of the other patients exhibited clinical or MRI signs of disease activity. In the EID4–6 cohort, no significant change was observed between the mean EDSS values at baseline and at 48 weeks (*p* = 0.68). Although three patients converted from RRMS to secondary progressive MS during follow-up, only one showed a significant increase in the EDSS. Of the other two patients, one showed an increase of 0.5 points in the EDSS, and the other showed no change in the EDSS, but experienced a progressive reduction in walking distance. Accordingly, overall, NEDA-3 was achieved in 66/70 patients (94%).

### sNfL concentrations compared between SID and EID treatment groups

**Figure 3.**
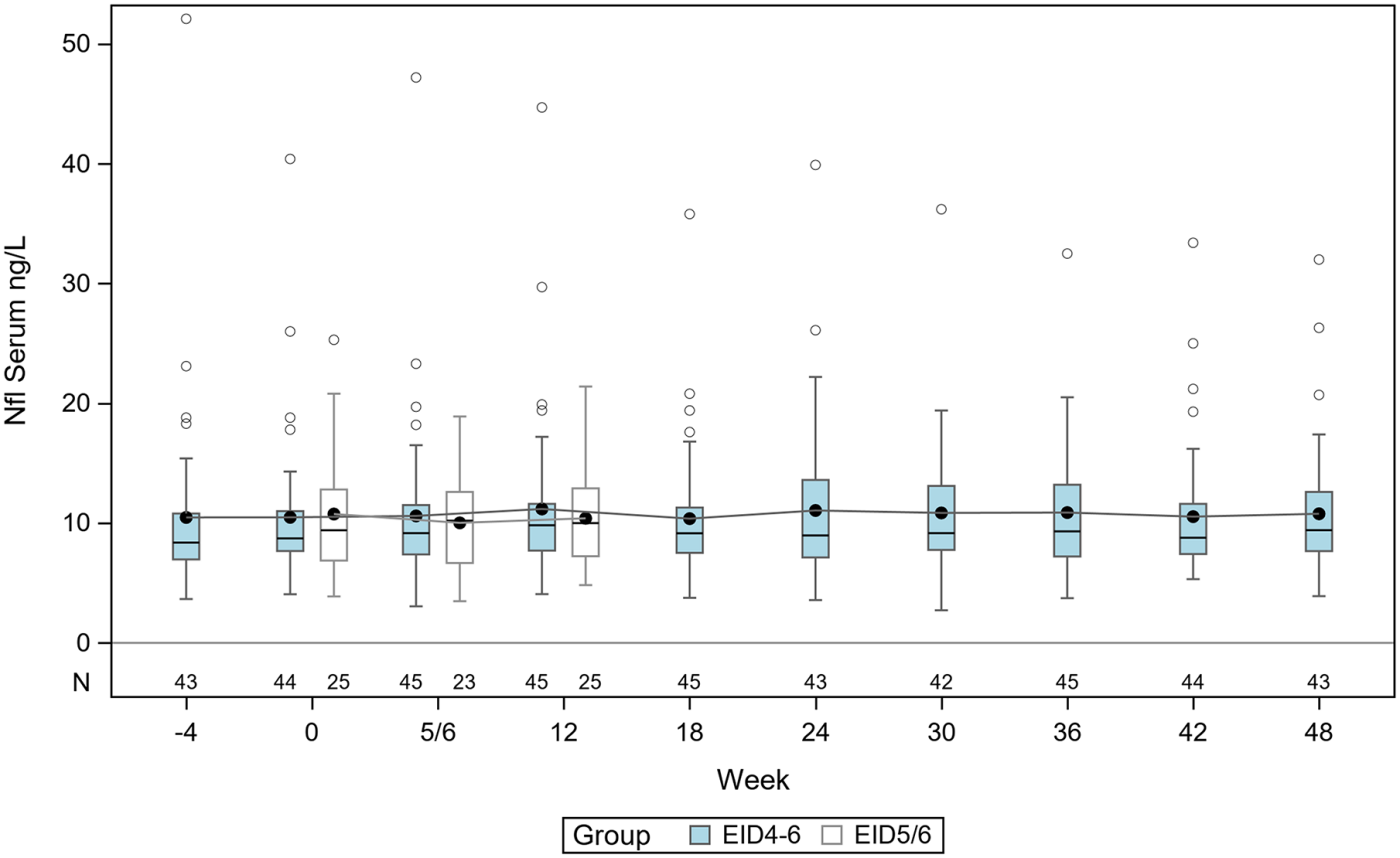
Serum NfL levels in ng/L in group EID4–6 (blue) and EID5/6 (white) where line in box is median and marker shows the mean. The boxes indicate the interquartile range and the vertically extending lines are minimum and maximum except for individual samples indicated as circles. *N* denotes the number of individuals in each group at each sampling.

In the EID4–6 cohort, the mean sNfL concentration at baseline (week zero) was 10.5 ng/L (standard deviation (SD) = 6.1) (i.e. before switching from the 4-week to the 6-week dosing interval). We compared changes in mean sNfL concentrations between all samples, but also between baseline and the early period (weeks 6–18), and between baseline and the entire study period (weeks 6–48). Serum NfL concentrations remained stable throughout the 48-week study period with no significant change in the mean sNfL at any time-point (Figure 3).

In the EID5/6 cohort, the mean sNfL concentration at baseline was 10.8 ng/L (SD = 5.3) and for all samples 10.3 ng/L (SD = 4.2), which was of similar level as the mean sNfL in the EID4–6 cohort (10.5 ng/L; SD = 6.1) before extending the NZ dosing interval (baseline). There was no statistically significant difference in the baseline mean sNfL between EID4–6 and EID5/6 (−0.28 ng/L, 95% confidence interval (CI) = −2.97–2.70). Hence, we found no significant increase of sNfL in NZ-treated patients with EID compared with SID. Furthermore, the sNfL concentrations did not change significantly in the patient who experienced a relapse and a non-enhancing new lesion or in patients who converted to secondary progressive MS.

### Inter- and intra-individual variation of sNfL in NZ-treated RRMS

In the EID4-6 cohort, we investigated the inter- and intra-individual variation of sNfL in order to evaluate sNfL as a biomarker for individual patients. The mean sNfL (all samples) was 10.8 ng/L (SD 5.9), and the median sNfL 8.9 ng/L (range = 4.5–39.4). We found that sNfL concentrations varied significantly with age (*R* = 0.48, *p* < 0.001), but not with BMI (Spearman’s correlation value; −0.18; *p* = 0.15), EDSS (*p* = 0.243) or gender (*p* = 0.979). After adjusting for age, the mean sNfL (all samples) was 11 ng/L (95% CI = 9.6–12.3).

**Figure 4.**
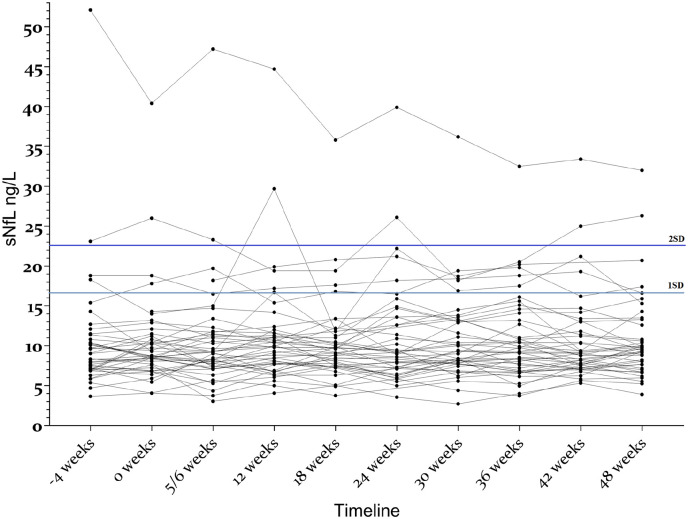
Variations in individual sNfL concentrations over time in patients with relapsing-remitting MS treated with extended-interval natalizumab dosing (EID). All patients were in the EID4–6 group. Dotted horizontal lines represent 1 (light blue) and 2 (dark blue) standard deviations (SD) above the mean.

The sNfL variability in EID4–6 was low, as illustrated in Figure 4. The mean individual SD (age adjusted) was 1.55 ng/L (SD = 1.12), and the mean age adjusted individual range was 4.9 ng/L (95% CI = 3.92–5.88).

We set the mean + 1 SD (16.7 ng/L) and the mean + 2 SD (22.6 ng/L) as cut offs for abnormality. We found one or more samples above this limit in six patients (47 samples, 10.5%) and three patients (17 samples, 3.8%), respectively. Furthermore, in three patients, all samples were above 1 SD, and in one patient, all samples were above 2 SD. None of these patients had clinical or MRI signs of disease activity or disease progression, but all were within the oldest age quartile (49–73 years) of the EID4–6 group. All patients with sNfL concentrations under or equal to 16.7 ng/L (i.e. 1 SD above the mean) were 48 years or younger. In contrast, 6/13 (46%) older patients had one or more samples with sNfL concentrations above 16.7 ng/L (Supplementary Figure S1).

One patient had remarkably high sNfL levels, with a declining trend, but no signs of clinical disease activity on MRIs or evidence of progression in the medical records.

### Serum-plasma NfL

**Figure 5.**
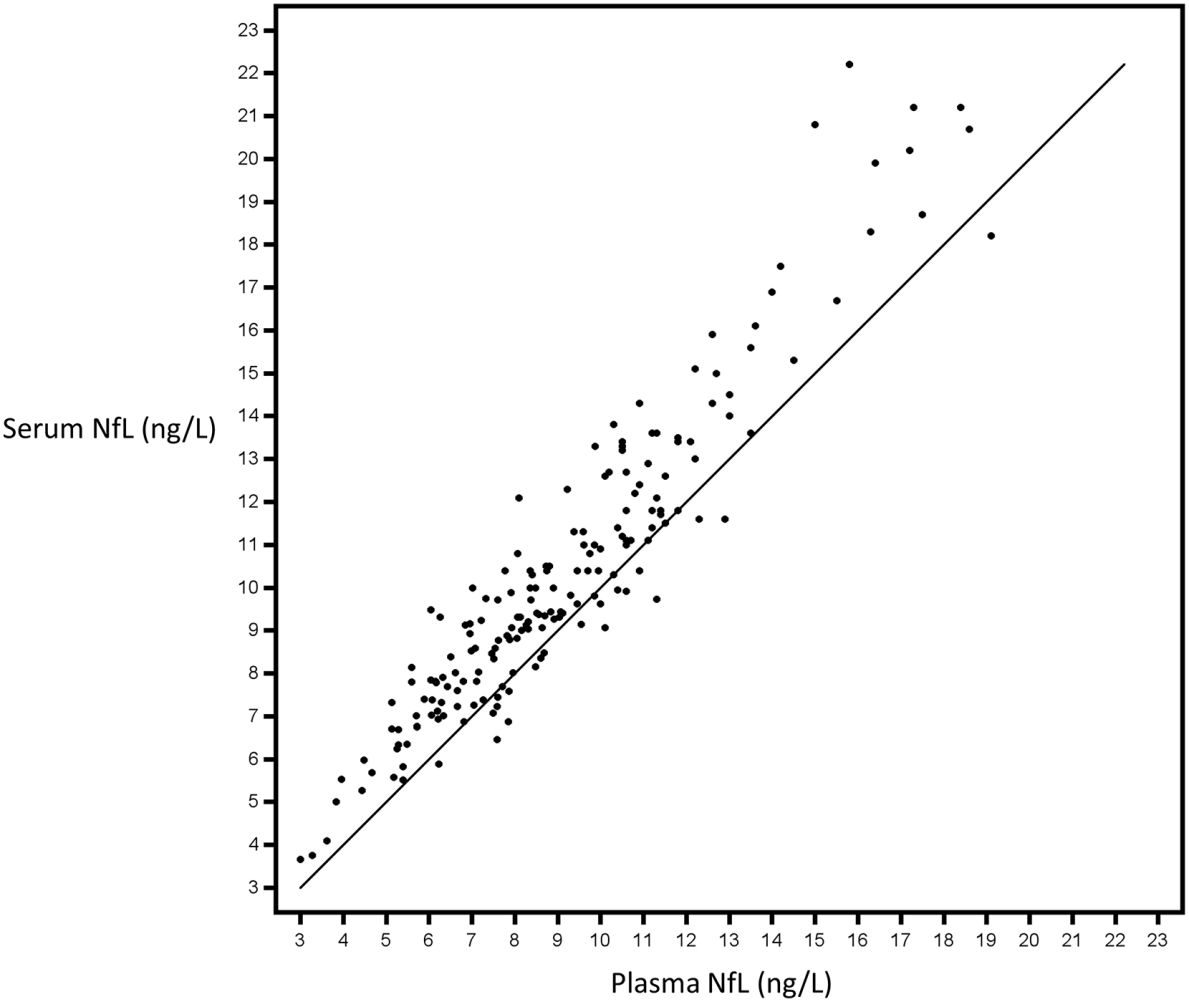
Paired serum and plasma neurofilament light chain (NfL) levels measured in a randomly selected subgroup of patients with relapsing-remitting MS who switched from standard-interval to extended-interval natalizumab dosing (EID). All patients (*N* = 19) were in the EID4–6 treatment group. Data are from 184 samples. The Shrout-Fleiss reliability random test showed an intra-class correlation coefficient of 0.88; Pearson’s correlation coefficient is *R* = 0.94 (*p* = 0.001).

Both sNfL and pNfL were collected from a randomly selected subgroup of the EID4–6 cohort (*N* = 19). The levels of sNfL and pNfL were highly correlated (*R* = 0.94; *p* < 0.001; intra-class correlation coefficient = 0.88). Figure 5 shows a pairwise comparison of sNfL and pNfL concentrations in individual samples. On average, the mean sNfL (10.4 ng/L, SD = 3.6) was 14% higher than the mean pNfL (9.13 ng/L, SD = 3.11; *p* < 0.001).

## Discussion

The results from this study were consistent with those from previous studies, which showed that extending NZ dosing from 4 to 6 weeks did not affect clinical or MRI measures.^6–10^ In addition, we showed that the sNfL concentrations were unchanged during 12 months of EID with NZ in patients with RRMS. The NfL concentrations in patients who received SID were similar to those observed in patients who had received EID prior to baseline. These findings supported the notion that axonal damage, determined with sNfL, did not increase when patients were switched to EID with NZ.

The SID of 4 weeks was based on pharmacokinetic and pharmacodynamic properties of NZ. With SID, NZ concentrations are maintained at levels that ensure at least 70%–80% continuous saturation of α4β1 integrin receptors.^30^

However, several studies have shown that much lower NZ receptor occupancy was sufficient to block the extravasation of autoreactive immune cells,^31,32^ the culprit of the CNS attack in RRMS. Previous studies have shown that interruptions longer than 12 weeks in NZ treatment led to increased risk of disease activity.^31–35^ In contrast, the efficacy provided with 5- to 8-week dosing intervals appeared to be similar to that achieved with SID, when evaluating relapse rate, EDSS, and lesions detected with MRI.^6–11,36^

However, inflammatory activity and degeneration may escape detection with conventional monitoring.^12–14^ We have previously shown that NfL and CXCL13 in CSF may be increased in patients without clinical or on MRI signs of disease activity or progression.^14^ Moreover, patients with clinically stable RRMS may experience disability deterioration and/or slowly expanding lesions.^13^

Even though smoldering MS activity may escape detection by conventional monitoring of RRMS,^12^ paramagnetic rim MRI lesions (PRLs) have been associated with chronic or smoldering lesions.^13^ Recently, increased sNfL levels were found in RRMS and progressive MS without recent disease activity but with two or more PRLs.^37^ Thus, although smoldering MS is considered a slow process, it may give rise to elevations in sNfL levels. In the same study, PRLs were associated with disease severity. In contrast, this study population included only patients with RRMS at baseline and with a few exceptions they did not progress at follow-up. Besides, the observational period was limited to 12 months which probably was too short to detect increases of sNfL due to smoldering MS. However, we cannot rule out that EID with NZ can impact such disease activity.

We monitored sNfL levels to detect new disease activity in patients treated with NZ that switched from SID to EID. Previous studies have used sNfL to evaluate potential disease recurrences in patients who switched from SID to EID. In one previous study, 34 patients with RRMS switched from SID to EID (from 5- to 7-week intervals), and sNfL was essentially unchanged from baseline, after 12 months of NZ treatment.^8^ In another study, sNfL remained stable for up to 8 weeks after discontinuing NZ, due to JC virus antibody positivity, but increased sNfL levels were detected at follow-up.^38^ In a group of 60 stable MS patients on SID NZ treatment, no change in sNfL was seen at 6 months after switch to ⩾35 days dosing interval.^39^ However, our study was the first to monitor sNfL at a relatively high frequency (every 6 weeks) for an extended period of time (48 weeks).

Repeated serum sampling provided the means to investigate treatment effects at both the individual and group levels over time. Due to the temporal change of NfL after a relapse or CNS injury, the interval between testing sNfL should not exceed 3–6 months.^20,40^ When we determined sNfL concentrations every 6 weeks, we found that most NZ-treated patients had only minor fluctuations of sNfL. We also found that sNfL concentrations were slightly, but significantly higher than pNfL concentrations, consistent with findings from a previous study.^41^ With a few exceptions, included patients had essentially no disease activity or disability progression and 94% achieved NEDA-3. Although this selected cohort had apparently very low disease activity prior baseline and during the 48-week study period, approximately 11% and 4% of samples had sNfL concentrations greater than 1 SD and 2 SD, respectively, above the mean sNfL. However, most of those samples were obtained from a few patients who had relatively high sNfL levels during the entire study period. In these patients, the elevated sNfL levels were not associated with disease activity or progression. Among the six patients with sNfL values above 1 SD, two had diabetes, which is known to influence sNfL levels,^42^ and all six patients were within the oldest quartile of the EID4–6 group. Similarly, in a previous study, the sNfL variance was higher among individuals of older age, in a population of patients with RRMS who had been followed with repeated serum sampling.^43^ The sNfL inter-individual variability seemed larger than the intra-individual variability, suggesting that the best utility of sNfL measurements is for individual longitudinal follow-up of younger adult age. Nevertheless, in our cohorts, some patients displayed unexplained variations in sNfL levels, which suggested the possibility that subtle disease activity could have occurred, but was not detected with conventional monitoring.^12^

This study had some limitations. One limitation was the relatively small number of patients included, preventing us from establishing a valid age- and BMI-adjusted reference sNfL concentration in stable RRMS. Although some population-based surveys of healthy subjects have provided age- and BMI-adjusted sNfL concentrations,^41,43^ there is an unmet need for data on sNfL variability in clinically meaningful subgroups of patients with MS. Perhaps the most important such cohort would be patients with RRMS that receive disease-modifying treatment, particularly those with stable disease monitored with conventional clinical and MRI measures. Our results from repeated sNfL determinations, in most cases, confirmed the stability of clinical and MRI measurements, but individual deviations could occur. A second limitation was that, in contrast to previous studies,^19,20,44^ we identified new disease activity in only one patient. Thus, we could not investigate the utility of sNfL testing for detecting disease activity in our patient cohort. Another limitation was the lack of healthy control subjects to serve as a reference for comparing sNfL levels to those found in patients with stable disease under NZ treatment. However, the sNfL levels of this study population were similar to those observed in healthy control subjects in a previous study of ours, using similar Simoa assay,^17^ but slightly higher than those reported in other studies.^41,43^

In conclusion, based on repeated determinations of sNfL measurements over 48 weeks, we did not find any signs of increased axonal damage in patients who received NZ and switched from SID to EID. Our data supported the notion that sNfL monitoring was most reliable for monitoring younger adult patients with RRMS, while increased sNfL concentrations may still occur in stable RRMS where confounding factors such as comorbidities are more common.

## Supplemental Material

sj-tif-1-msj-10.1177_13524585221108080 – Supplemental material for No increase of serum neurofilament light in relapsing-remitting multiple sclerosis patients switching from standard to extended-interval dosing of natalizumab Click here for additional data file. Supplemental material, sj-tif-1-msj-10.1177_13524585221108080 for No increase of serum neurofilament light in relapsing-remitting multiple sclerosis patients switching from standard to extended-interval dosing of natalizumab by Magnus Johnsson, Helen H Farman, Kaj Blennow, Henrik Zetterberg, Clas Malmeström, Markus Axelsson and Jan Lycke in Multiple Sclerosis Journal

## References

[1] EMA. Tysabri (natalizumab); an overview of Tysabri and why it is authorised in the EU, https://www.ema.europa.eu/en/documents/overview/tysabri-epar-medicine-overview_en.pdf

[2] Polman CH O’Connor PW Havrdova E , et al. A randomized, placebo-controlled trial of natalizumab for relapsing multiple sclerosis. N Engl J Med 2006; 354: 899–910. 16510744 10.1056/NEJMoa044397

[3] Ho PR Koendgen H Campbell N , et al. Risk of natalizumab-associated progressive multifocal leukoencephalopathy in patients with multiple sclerosis: A retrospective analysis of data from four clinical studies. Lancet Neurol 2017; 16(11): 925–933. 28969984 10.1016/S1474-4422(17)30282-X

[4] Biogen. Global natalizumab (TYSABRI) postmarketing PML update. Cambridge, MA: Biogen, 2021.

[5] Ryerson LZ Foley J Chang I , et al. Risk of natalizumab-associated PML in patients with MS is reduced with extended interval dosing. Neurology 2019; 93: e1452–e1462. 10.1212/WNL.0000000000008243 PMC7010325 31515290

[6] Zhovtis Ryerson L Frohman TC Foley J , et al. Extended interval dosing of natalizumab in multiple sclerosis. J Neurol Neurosurg Psychiatry 2016; 87: 885–889. 26917698 10.1136/jnnp-2015-312940

[7] Bomprezzi R Pawate S . Extended interval dosing of natalizumab: A two-center, 7-year experience. Ther Adv Neurol Disord 2014; 7(5): 227–231. 25342976 10.1177/1756285614540224 PMC4206618

[8] van Kempen ZLE Hoogervorst ELJ Wattjes MP , et al. Personalized extended interval dosing of natalizumab in MS: A prospective multicenter trial. Neurology 2020; 95: e745–e754. 10.1212/WNL.0000000000009995 32690785

[9] Clerico M De Mercanti SF Signori A , et al. Extending the interval of natalizumab dosing: Is efficacy preserved? Neurotherapeutics 2020; 17(1): 200–207. 31452081 10.1007/s13311-019-00776-7 PMC7007494

[10] Foley JF Defer G Ryerson LZ , et al. Comparison of switching to 6-week dosing of natalizumab versus continuing with 4-week dosing in patients with relapsing-remitting multiple sclerosis (NOVA): A randomised, controlled, open-label, phase 3b trial. Lancet Neurol. Epub ahead of print 25 April 2022. DOI: 10.1016/S1474-4422(22)00143-0. 35483387

[11] Chisari CG Grimaldi LM Salemi G , et al. Clinical effectiveness of different natalizumab interval dosing schedules in a large Italian population of patients with multiple sclerosis. J Neurol Neurosurg Psychiatry 2020; 91(12): 1297–1303. 33055141 10.1136/jnnp-2020-323472

[12] Giovannoni G Popescu V Wuerfel J , et al. Smouldering multiple sclerosis: The “real MS.” Ther Adv Neurol Disord 2022; 15: 17562864211066751. 10.1177/17562864211066751 PMC8793117 35096143

[13] Absinta M Sati P Masuzzo F , et al. Association of chronic active multiple sclerosis lesions with disability in vivo. JAMA Neurol 2019; 76: 1474–1483. 31403674 10.1001/jamaneurol.2019.2399 PMC6692692

[14] Novakova L Axelsson M Malmeström C , et al. NFL and CXCL13 may reveal disease activity in clinically and radiologically stable MS. Mult Scler Relat Disord 2020; 46: 102463. 32862040 10.1016/j.msard.2020.102463

[15] Lycke J Zetterberg H . The role of blood and CSF biomarkers in the evaluation of new treatments against multiple sclerosis. Expert Rev Clin Immunol 2017; 13(12): 1143–1153. 29090607 10.1080/1744666X.2017.1400380

[16] Rosenstein I Axelsson M Novakova L , et al. Exploring CSF neurofilament light as a biomarker for MS in clinical practice; a retrospective registry-based study. Mult Scler 2022; 28(6): 872–884. 34392718 10.1177/13524585211039104 PMC9024026

[17] Novakova L Zetterberg H Sundstrom P , et al. Monitoring disease activity in multiple sclerosis using serum neurofilament light protein. Neurology 2017; 89: 2230–2237. 29079686 10.1212/WNL.0000000000004683 PMC5705244

[18] Kuhle J Barro C Andreasson U , et al. Comparison of three analytical platforms for quantification of the neurofilament light chain in blood samples: ELISA, electrochemiluminescence immunoassay and Simoa. Clin Chem Lab Med 2016; 54: 1655–1661. 27071153 10.1515/cclm-2015-1195

[19] Norgren N Sundström P Svenningsson A , et al. Neurofilament and glial fibrillary acidic protein in multiple sclerosis. Neurology 2004; 63: 1586–1590. 15534240 10.1212/01.wnl.0000142988.49341.d1

[20] Lycke JN Karlsson JE Andersen O , et al. Neurofilament protein in cerebrospinal fluid: A potential marker of activity in multiple sclerosis. J Neurol Neurosurg Psychiatry 1998; 64(3): 402–404. 9527161 10.1136/jnnp.64.3.402 PMC2170011

[21] Gunnarsson M Malmeström C Axelsson M , et al. Axonal damage in relapsing multiple sclerosis is markedly reduced by natalizumab. Ann Neurol 2011; 69(1): 83–89. 21280078 10.1002/ana.22247

[22] Delcoigne B Manouchehrinia A Barro C , et al. Blood neurofilament light levels segregate treatment effects in multiple sclerosis. Neurology 2020; 94: e1201–e1212. 10.1212/WNL.0000000000009097 PMC7387108 32047070

[23] Thompson AJ Banwell BL Barkhof F , et al. Diagnosis of multiple sclerosis: 2017 revisions of the McDonald criteria. Lancet Neurol 2018; 17: 162–173. 29275977 10.1016/S1474-4422(17)30470-2

[24] Kurtzke JF . Rating neurologic impairment in multiple sclerosis: An expanded disability status scale (EDSS). Neurology 1983; 33(11): 1444–1452. 6685237 10.1212/wnl.33.11.1444

[25] Lechner-Scott J Kappos L Hofman M , et al. Can the Expanded Disability Status Scale be assessed by telephone? Mult Scler 2003; 9: 154–159. 12708811 10.1191/1352458503ms884oa

[26] Vågberg M Axelsson M Birgander R , et al. Guidelines for the use of magnetic resonance imaging in diagnosing and monitoring the treatment of multiple sclerosis: Recommendations of the Swedish Multiple Sclerosis Association and the Swedish Neuroradiological Society. Acta Neurol Scand 2017; 135(1): 17–24. 27558404 10.1111/ane.12667 PMC5157754

[27] Giovannoni G Turner B Gnanapavan S , et al. Is it time to target no evident disease activity (NEDA) in multiple sclerosis? Mult Scler Relat Disord 2015; 4(4): 329–333. 26195051 10.1016/j.msard.2015.04.006

[28] Collingridge DS . A primer on quantitized data analysis and permutation testing. J Mix Method Res 2013; 7: 81–97.

[29] Rousson V . Assessing inter-rater reliability when the raters are fixed: Two concepts and two estimates. Biom J 2011; 53(3): 477–490. 21425184 10.1002/bimj.201000066

[30] Muralidharan KK Kuesters G Plavina T , et al. Population pharmacokinetics and target engagement of natalizumab in patients with multiple sclerosis. J Clin Pharmacol 2017; 57(8): 1017–1030. 28398628 10.1002/jcph.894

[31] Derfuss T Kovarik JM Kappos L , et al. α4-integrin receptor desaturation and disease activity return after natalizumab cessation. Neurol Neuroimmunol Neuroinflamm 2017; 4(5): e388. 10.1212/NXI.0000000000000388 PMC5572051 28856176

[32] Plavina T Muralidharan KK Kuesters G , et al. Reversibility of the effects of natalizumab on peripheral immune cell dynamics in MS patients. Neurology 2017; 89: 1584–1593. 28916537 10.1212/WNL.0000000000004485 PMC5634662

[33] O’Connor PW Goodman A Kappos L , et al. Disease activity return during natalizumab treatment interruption in patients with multiple sclerosis. Neurology 2011; 76: 1858–1865. 21543733 10.1212/WNL.0b013e31821e7c8a

[34] Kaufman M Cree BA De Sèze J , et al. Radiologic MS disease activity during natalizumab treatment interruption: Findings from RESTORE. J Neurol 2015; 262(2): 326–336. 25381458 10.1007/s00415-014-7558-6

[35] Trojano M Ramió-Torrentà L Grimaldi LM , et al. A randomized study of natalizumab dosing regimens for relapsing–remitting multiple sclerosis. Mult Scler 2021; 27(14): 2240–2253. 33821693 10.1177/13524585211003020 PMC8597184

[36] Butzkueven H Kappos L Spelman T , et al. No evidence for loss of natalizumab effectiveness with every-6-week dosing: A propensity score-matched comparison with every-4-week dosing in patients enrolled in the Tysabri Observational Program (TOP). Ther Adv Neurol Disord 2021; 14: 17562864211042458. 10.1177/17562864211042458 PMC8481711 34603507

[37] Maggi P Kuhle J Schädelin S , et al. Chronic white matter inflammation and serum neurofilament levels in multiple sclerosis. Neurology 2021; 97: e543–e553. 10.1212/WNL.0000000000012326 PMC8424501 34088875

[38] Proschmann U Inojosa H Akgün K , et al. Natalizumab pharmacokinetics and -dynamics and serum neurofilament in patients with multiple sclerosis. Front Neurol 2021; 12: 650530. 33935948 10.3389/fneur.2021.650530 PMC8079654

[39] Foley J Xiong K Hoyt T , et al. Serum neurofilament light (sNfL) levels in patients with relapsing-remitting multiple sclerosis (RRMS) switching from natalizumab every-4-week (Q4W) dosing to extended interval dosing (EID) (2013). Neurology 2020; 94: 2013.

[40] Malmeström C Haghighi S Rosengren L , et al. Neurofilament light protein and glial fibrillary acidic protein as biological markers in MS. Neurology 2003; 61: 1720–1725. 14694036 10.1212/01.wnl.0000098880.19793.b6

[41] Benkert P Meier S Schaedelin S , et al. Serum neurofilament light chain for individual prognostication of disease activity in people with multiple sclerosis: A retrospective modelling and validation study. Lancet Neurol 2022; 21(3): 246–257. 35182510 10.1016/S1474-4422(22)00009-6

[42] Mariotto S Carta S Bozzetti S , et al. Sural nerve biopsy: Current role and comparison with serum neurofilament light chain levels. J Neurol 2020; 267(10): 2881–2887. 32462349 10.1007/s00415-020-09949-3

[43] Khalil M Pirpamer L Hofer E , et al. Serum neurofilament light levels in normal aging and their association with morphologic brain changes. Nat Commun 2020; 11: 812. 32041951 10.1038/s41467-020-14612-6 PMC7010701

[44] Calabresi PA Arnold DL Sangurdekar D , et al. Temporal profile of serum neurofilament light in multiple sclerosis: Implications for patient monitoring. Mult Scler 2021; 27(10): 1497–1505. 33307998 10.1177/1352458520972573 PMC8414824

