# Supplementary figures and images for: No increase of serum neurofilament light in relapsing-remitting multiple sclerosis patients switching from standard to extended-interval dosing of natalizumab
Source: Mult Scler. 2022 Jul 20;28(13):2070–80. doi: 10.1177/13524585221108080 (PMC9574231; doi:10.1177/13524585221108080)

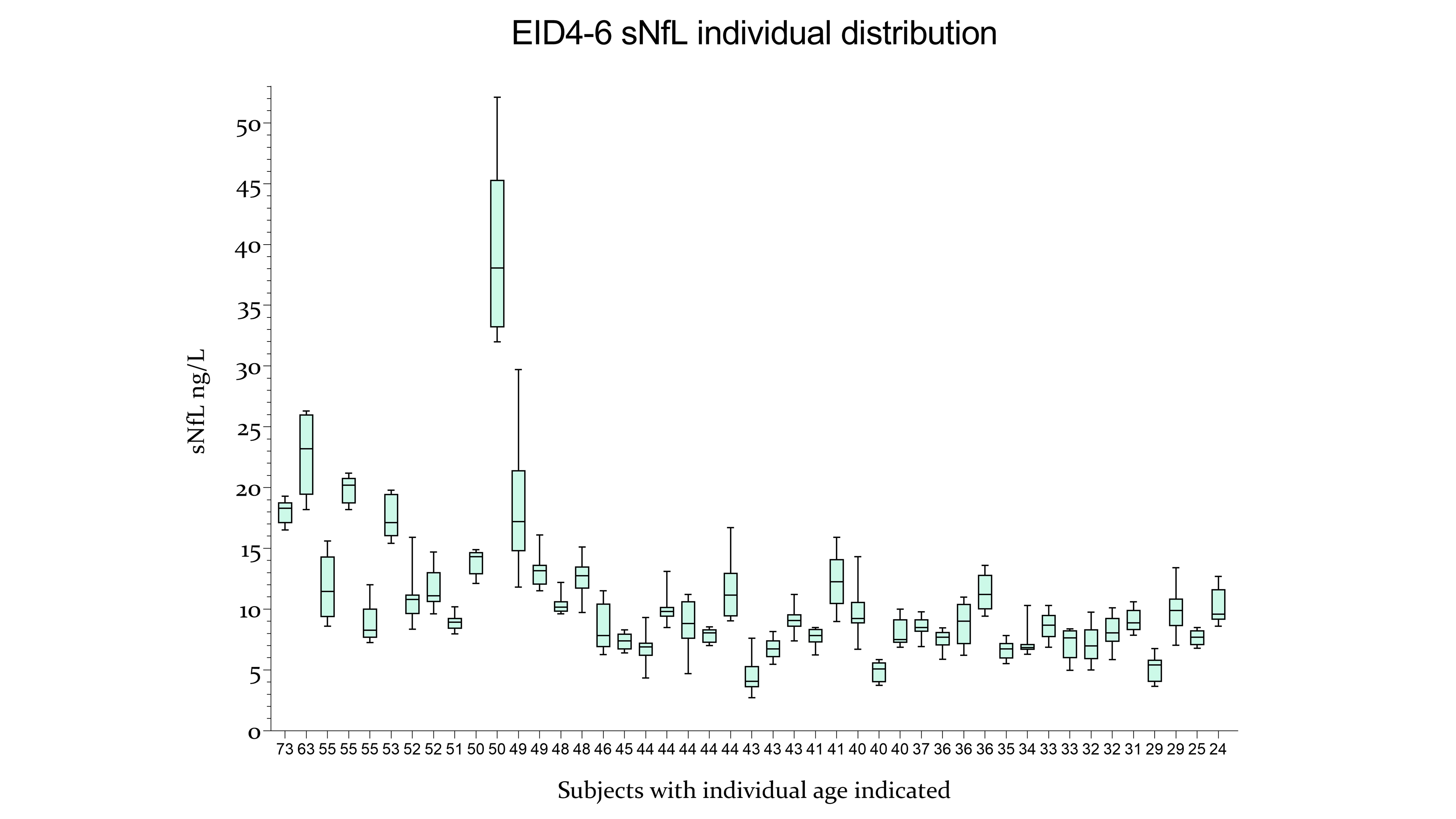

Supplement: sj-tif-1-msj-10.1177_13524585221108080 – Supplemental material for No increase of serum neurofilament light in relapsing-remitting multiple sclerosis patients switching from standard to extended-interval dosing of natalizumab [file sj-tif-1-msj-10.1177_13524585221108080.tif]
